# Supplementary material for: Spatio-temporal predictive modeling framework for infectious disease spread
Source: Sci Rep. 2021 Mar 24;11:6741. doi: 10.1038/s41598-021-86084-7 (PMC7990963; doi:10.1038/s41598-021-86084-7)
Supplement: Supplementary file 1 — Supplementary Information 1. [file 41598_2021_86084_MOESM1_ESM.pdf]

Spatio-temporal predictive modeling framework for infectious disease spread

Sashikumaar Ganesan, Deepak Subramani
Department of Computational and Data Sciences,
Indian Institute of Science Bangalore 560012

Supplementary Materials

Numerical Scheme

Let $T_\infty = 365$ days, $d_\infty = 400$ days, $a_\infty = 45,625$ days and $\Omega_{\rm x} := \cup\Omega_k$, $k = 1, \dots, M$, where M being the number of states and union territories in India. We assume that there will be no inter- or intra-state and international movements, that is, $\mathbf{u} = 0$, no growth in level of infection, that is, $G_{\ell_v} = 0$, the growth age is negligible and no source. Nevertheless, population distribution in all three internal ordinates ℓ_v, ℓ_d and ℓ_a are described and tracked. Hence, the PBE in model becomes

$$\frac{\partial I}{\partial t} + \frac{\partial I}{\partial \ell_d} + CI = 0 \quad \text{in} \quad (0, \mathrm{T}_\infty] \times \Omega_{\rm x} \times \Omega_\ell. \tag{1}$$

Operator splitting finite element scheme

A finite element scheme [5] based on operator splitting [2, 3, 4] is used to solve the high-dimensional PBE model (1). Applying, operator splitting to (1), we get

Step 1. (x-direction) For given $I(t^a, x, \ell)$ with $I(t^a = 0, x, \ell) = I_0$, find $\tilde{I}(t^b, x, \ell)$ in (t^a, t^b) for all $\ell \in \Omega_\ell$ such that

$$\frac{\partial \tilde{I}}{\partial t} + CI = 0, \quad \tilde{I}(t^a, x, \ell) = I(t^a, x, \ell) \quad \text{in} \quad \Omega_{\rm x}\,, \tag{2}$$

Step 2. (ℓ_d-direction) For given $\tilde{I}(t^b, x, \ell)$, find $I(t^b, x, \ell)$ in (t^a, t^b) for all $x \in \Omega_{\rm x}$ $\ell_v \in L_v$ and $\ell_a \in L_a$ such that

$$\frac{\partial I}{\partial t} + \frac{\partial I}{\partial \ell_d} = 0, \quad I(t^a, x, \ell) = \tilde{I}(t^b, x, \ell) \quad \text{in} \quad \Omega_\ell\,; \qquad \mathrm{I(t, \ell_v, 0, \ell_a) = B_{nuc} \quad in \quad L_a}, \tag{3}$$

In the x-direction, the evaluation equation (2) has to be solved for every $\ell \in \Omega_\ell$ by considering ℓ as a parameter. Similarly, the system (3) has to be solved in ℓ_d-direction for every $x \in \Omega_{\rm x}$ $\ell_v \in L_v$ and $\ell_a \in L_a$ by considering these variables as parameters. The backward Euler and discontinues Galerkin with upwind methods are used for the temporal and the spatial discretization, respectively. The implementation of the splitting algorithm in the finite element context has been presented in these papers [2, 3, 4].

Parameters for Covid-19 predictions

The nucleation model $B_{\rm nuc}$ defined in the result section is considered with $R_0 = 3.35$, $f_1 = 1$, $f_2 = 1$, $f_4 = 1$ and

$$f_3(t, S_D) = 1. - 1./(1. + \exp(-(S_D(t) - 0.5)/0.1)), \quad S_D(t) = \begin{cases} 0.7 + 0.001333\, t & 0 \le t < 15 \\ 0.72 + 0.004285(t - 15) & 15 \le t < 36 \\ 0.81 - 0.004(t - 36) & 36 \le t < 51 \\ 0.75 + 0.0012(t - 51.) & 51 \le t < 72 \\ 0.775 + ds(t - 72) & \text{else} \end{cases}$$

Moreover, the values given in Table 1 are used for ds and f_1 to perform scenario analysis. Furthermore, the quarantine function

$$\gamma_Q(\ell_d) = \begin{cases} 1 & 0 \le t < 1 \\ 0.9(1./(1. + \exp(-(l_v - 0.4)/0.1))) * (1./(1. + \exp(-(l_d - 5.1)/2))) & \text{else,} \end{cases}$$

Table 1: Parameter values used in scenario analysis.

Scenarios	Current Trend	Better Trend	Worse-case	Periodic lockdowns
ds	0.01	0.015	0.005	0.01 & $f_1 = 0.01$ on lockdown days

is used in all scenarios. Finally, the recovery and death rate functions are fitted as

$$C_{ID}(t, \ell_v) = \begin{cases} 0 & \ell_v < 0.64 \\ 0.0475 - 0.000357\,t & \ell_v \geq 0.64 \& t < 21 \\ 0.0475 + 0.000208(t-21) & \ell_v \geq 0.64 \& 21 \leq t < 36 \\ 0.0475 & \text{else,} \end{cases} \quad , \quad C_R(t) = \begin{cases} 0.01 + t * 0.00058 & t < 65 \\ 0.0475 & \text{else.} \end{cases}$$

Remark: A factor $N_S(t)/N(t)$ needs to be introduced in B_{nuc} when herd immunity develops.

Initial infection number density

For India, the data to estimate $N_D(\mathbf{x})$ is downloaded from a publicly sourced database [1]. To distribute the number density among the internal ordinates, we employ distribution fits as given in the result section. Data set of age downloaded from Statista [6] is used to fit a_1, b_1, c_1 in the definition of initial distribution.

References

[1] Crowdsourced. *COVID-19 India. https://www.covid19india.org/, accessed on April 10*, 2020.

[2] S. Ganesan. An operator-splitting Galerkin/SUPG finite element method for population balance equations: Stability and convergence. *ESAIM: M2AN*, 46:1447–1465, 2012.

[3] S. Ganesan and L. Tobiska. An operator-splitting finite element method for the efficient parallel solution of multidimensional population balance systems. *Chem. Eng. Sci.*, 69(1):59–68, 2012.

[4] S. Ganesan and L. Tobiska. Operator-splitting finite element algorithms for computations of high-dimensional parabolic problems. *Appl. Math. Comp.*, 219:6182–6196, 2013.

[5] S. Ganesan and L. Tobiska. *Finite Elements: Theory and Algorithms.* Cambridge IISc Series. Cambridge University Press, 2016.

[6] Statista. *Statista Website. https://www.statista.com/statistics/1110522/india-number-of-coronavirus-cases-by-age-group/*, 2020.
